# Supplementary material for: Analysis of MTNR1A Genetic Polymorphisms and Their Association with the Reproductive Performance Parameters in Two Mediterranean Sheep Breeds
Source: Animals (Basel). 2023 Jan 28;13(3):448. doi: 10.3390/ani13030448 (PMC9913639; doi:10.3390/ani13030448)
Supplement: Supplementary file 1 [file animals-13-00448-s001.zip › animals-2127669-supplementary.pdf]

Supplementary Table S1. Haplotypes and their frequency in all the ewes

Haplotype	SNP1	SNP2	SNP3	SNP4	SNP5	SNP6	SNP7	SNP8	SNP9	SNP10	SNP11	SNP12	SNP13	SNP14	SNP15	SNP16	SNP17	SNP18	SNP19	SNP20	SNP21	SNP22	SNP23	SNP24	SNP25	SNP26	Frequency	OR (95% CI)	P-value
h1	G	C	G	C	C	C	A	C	G	T	G	A	G	C	A	G	C	G	G	G	G	G	G	C	C	C	0,2961	1	---
h2	T	T	A	T	T	T	G	C	A	G	A	G	G	T	G	G	T	G	G	G	G	G	G	C	C	C	0,1039	10.01 (1.67 - 60.	0,014

																											05)	
h3	T	C	G	T	T	T	A	C	A	G	A	G	G	T	A	G	T	G	G	G	A	A	G	T	A	C	0,0909	0.87 (0.18 - 4.21)	0,86
h4	G	C	G	C	C	C	A	C	G	T	G	A	A	C	A	T	C	A	G	A	G	G	G	T	C	C	0,087	4.11 (0.78 - 21.	0,1

																												55)	
h5	T	C	G	T	T	C	A	C	A	G	A	G	G	T	A	G	T	G	G	G	A	A	G	T	A	C	0,0844	1.39 (0.28-6.97)	0,69
h6	G	C	G	C	C	C	A	C	G	T	G	A	G	C	A	G	T	G	A	G	G	G	G	C	C	C	0,0649	8.71 (1.18-64.	0,038

																												39)	
h7	T	T	A	T	T	C	A	C	A	G	A	G	G	T	G	G	T	G	G	G	G	G	G	C	C	C	0,0545	0.86 (0.06 - 11.82)	0,91
h8	G	C	G	C	C	C	A	C	G	T	G	A	G	C	A	G	T	G	G	G	G	G	A	C	C	C	0,0519	–	<0.0001
h9	T	T	A	T	T	T	G	C	A	G	A	G	G	T	G	T	C	A	G	A	G	G	G	T	C	C	0,026	–	<0.0001

h10	T	T	A	T	T	C	A	C	A	G	A	G	G	T	G	T	C	A	G	A	G	G	G	T	C	C	0,0195	0.73 (0.04-12.47)	0,833
h11	G	C	G	C	C	C	A	C	G	T	G	A	G	C	A	G	T	G	G	G	G	G	G	C	C	T	0,013	0	1
h12	T	C	G	T	C	C	A	C	A	G	A	G	G	T	A	G	T	G	G	G	A	A	G	T	A	C	0,013	0	1
h13	T	C	G	T	T	C	A	T	A	G	A	G	A	T	A	G	T	G	A	G	G	G	G	C	C	C	0,013	–	<0.0001

h14	T	T	A	T	T	C	G	C	A	G	A	G	G	T	G	T	C	A	G	A	G	G	G	T	C	C	0,013	4.08 (0.19 - 87.83)	0,37
h15	T	T	A	T	T	T	G	C	A	G	A	G	G	T	G	T	C	A	G	A	A	G	G	T	C	C	0,013	0	1

Supplementary Table S2. Results of the first year in the studied ewes from both breeds: DTL, fertility rate, and litter size for each genotype at SNP17.

SNP	SNP17			P
Genotype	T/T	T/C	C/C	

DTL (days)	208.215±23.590	215.407±27.228	198.500±18.299	n.s
Fertility rate (%)	100	100	100 %	n.s
Litter size	1.058±0.237	1.296±0.465	1.125±0.353	n.s

Supplementary Table S3. Results of the second year in the studied ewes from both breeds: DTL, fertility rate, and litter size for each genotype at SNP17.

SNP	SNP17			P
Genotype	T/T	T/C	C/C	
DTL (days)	204.827±20.918	215.250±25.898	208.500±16.266	P<0,05

Fertility rate (%)	100	100	100 %	n.s
Litter size	1.094±0.295	1.160±0.374	1.000±0.307	P<0,05

Supplementary Table S4. Results of the first year in the studied ewes from both breeds: DTL, fertility rate, and litter size for each genotype at SNP18/SNP20.

SNP	SNP18			P	SNP20			P
Genotype	G/G	G/A	A/A		G/G	G/A	A/A	
DTL (days)	207.283±23.539	211.090±21.480	201.000±17.981	n.s	207.283±23.539	211.090±21.480	201.000±17.981	n.s

Fertility rate (%)	100	100	100 %	n.s	100	100	100	n.s
Litter size	1.066±0.251	1.240±0.435	1.00±0.00	n.s	1.06±0.251	1.142±0.358	1.00±0.00	n.s

Supplementary Table S5. Results of the second year in the studied ewes from both breeds: DTL, fertility rate, and litter size for each genotype at SNP18/SNP20.

SNP	SNP18			P	SNP20			P
Genotype	G/G	G/A	A/A		G/G	G/A	A/A	
DTL (days)	209.866±23.903	207.181±17.206	222.500±11.269	P<0,05	209.866±23.903	207.181±17.206	222.500±11.27	P<0,05
Fertility rate (%)	100	100	100 %	n.s	100	100	100	n.s
Litter size	1.083±0.278	1.136±0.351	1.00±0.00	n.s	1.083±0.278	1.150±0.366	1.00±0.00	n.s

Supplementary Table S6. Results of lambs' birth weights for the three years in the studied ewes from both breed

Data are expressed as LSmeans.

SNP	SNP18			P	SNP20			P
Lambs birth weight	G/G	G/A	A/A		G/G	G/A	A/A	
2017	3.714±0.445	3.529±0.484	3.900±0.355	n.s	3.714±0.445	3.552±0.482	3.720±0.506	n.s
2018	3.641±0.699	3.452±0.472	3.900±0.648	n.s	3.641±0.699	3.408±0.428	4.020±0.622	n.s
2019	4.553±0.945	3.900±0.529	4.150±0.070	P<0,05	4.378±0.868	4.420±0.851	5.900±0.000	P<0,05

Supplementary Table S7. Results of lambs' birth weights for the three years in the studied ewes from both breed

Data are expressed as LSmeans.

SNP	SNP17			P
Lambs birth weight	T/T	T/C	C/C	
2017	2.674±0.864	2.951±0.897	3.025±0.686	n.s
2018	2.758±0.924	2.881±0.797	3.562±0.992	P<0,05
2019	3.052±1.122	3.563±1.094	4.112±1.124	P<0,05

Supplementary Table S8. Results of the first year in the studied Barbarine ewes: DTL, fertility rate, litter size and lambs birth weights for each genotype at SNP18/SNP20.

Data are expressed as LSmeans.

SNP	SNP18	P	SNP20	P

Genotype	G/G	G/A	A/A		G/G	G/A	A/A	
DTL (days)	200.9±17.808	203.545±12.832	213.000±19.798	P<0,05	200.9±17.808	203.545±12.832	213.000±19.798	P<0,05
Fertility rate (%)	100	100	100	n.s	100	100	100	n.s
Litter size	1.00±0.00	1.00±0.00	1.00±0.00	n.s	1.00±0.00	1.00±0.00	1.00±0.00	n.s

Supplementary Table S9. Results of the second year in the studied Barbarine ewes: DTL, fertility rate, litter size and lambs birth weights for each genotype at SNP18/SNP20.

Data are expressed as LSmeans.

SNP	SNP18			P	SNP20			P
Genotype	G/G	G/A	A/A		G/G	G/A	A/A	

DTL (days)	208.633±26.711	208.090±13.838	214.500±10.606	n.s	208.633±26.711	208.090±13.838	214.500±10.606	n.s
Fertility rate (%)	100	100	100	n.s	100	100	100	n.s
Litter size	1.066±0.253	1.00±0.00	1.00±0.00	n.s	1.066±0.253	1.00±0.00	1.00±0.00	n.s

Supplementary Table S10. Results of the first year in the studied ewes from Barbarine breed: DTL, fertility rate, and litter size for each genotype at SNP17.

SNP	SNP17			P
Genotype	T/T	T/C	C/C	
DTL (days)	211.875±12.182	198.125±18.518	201.421±15.510	n.s
Fertility rate (%)	100	100	100 %	n.s

Litter size	1.000±0.000	1.000±0.000	1.000±0.000	n.s

Supplementary Table S11. Results of the second year in the studied ewes from Barbarine breed: DTL, fertility rate, and litter size for each genotype at SNP17.

SNP	SNP17			P
Genotype	T/T	T/C	C/C	
DTL (days)	192.000±9.227	223.312±30.377	203.578±11.072	n.s
Fertility rate (%)	100	100	100 %	n.s
Litter size	1.125±0.353	1.000±0.000	1.052±0.229	n.s

Supplementary Table S12. Results of the first year in the studied Queue Fine de l'Ouest ewes: DTL, fertility rate, litter size and lambs birth weights for each genotype at SNP18/SNP20.

Data are expressed as LSmeans.

SNP	SNP18			P	SNP20			P
Genotype	G/G	G/A	A/A		G/G	G/A	A/A	
DTL (days)	213.666±26.942	218.636±26.058	189.000±1.414	n.s	213.666±26.942	213.800±21.647	215.000±45.044	n.s
Fertility rate (%)	100	100	100	n.s	100	100	100	n.s
Litter size	1.00±0.00	1.00±0.00	1.00±0.00	n.s	1.133±0.345	1.300±0.483	1.000±0.00	n.s

Supplementary Table S13. Results of the second year in the studied Queue Fine de l'Ouest ewes: DTL, fertility rate, litter size and lambs birth weights for each genotype at SNP18/SNP20.

Data are expressed as LSmeans.

SNP	SNP18			P	SNP20			P
Genotype	G/G	G/A	A/A		G/G	G/A	A/A	
DTL (days)	210.800±20.705	206.272±20.698	230.500±3.530	n.s	211.100±21.114	207.500±21.391	218.333±21.221	n.s
Fertility rate (%)	100	100	100	n.s	100	100	100	n.s
Litter size	1.100±0.305	1.272±0.467	1.000±0.00	n.s	1.100±0.305	1.300±0.483	1.000±0.00	n.s

Supplementary Table S14. Results of the first year in the studied ewes from QFO breed: DTL, fertility rate, and litter size for each genotype at SNP17.

SNP	SNP17			P
Genotype	T/T	T/C	C/C	
DTL (days)	207.619±18.869	224.312±30.240	207.333±33.678	n.s
Fertility rate (%)	100	100	100 %	n.s
Litter size	1.142±0.358	1.250±0.447	1.000±0.000	n.s

Supplementary Table S15. Results of the second year in the studied ewes from QFO breed: DTL, fertility rate, and litter size for each genotype at SNP17.

SNP	SNP17	P

Genotype	T/T	T/C	C/C	
DTL (days)	209.714±22.179	207.187±18.019	224.000±21.175	n.s
Fertility rate (%)	100	100	100 %	n.s
Litter size	1.047±0.218	1.312±0.478	1.000±0.000	P<0,05

Supplementary Table S16. Results of lambs birth weights for the three years in the studied Barbarine and QFO ewes breed

Data are expressed as LSmeans.

SNP	SNP18			P	SNP20			P
Genotype	G/G	G/A	A/A		G/G	G/A	A/A	

Lambs birth weights									
Barbarine ewes	2017	3.813±0.394	3.827±0.355	4.150±0.070	n.s	3.813±0.394	3.827±0.355	4.150±0.070	n.s
	2018	3.653±0.867	3.372±0.363	3.750±1.060	n.s	3.653±0.867	3.372±0.363	3.750±1.060	n.s
	2019	4.670±1.107	4.700±1.019	6.500±0.721	P<0,05	4.670±1.107	4.700±1.019	6.500±0.721	P<0,05
QFO ewes	2017	3.624±0.475	3.276±0.441	3.650±0.353	n.s	3.624±0.475	3.276±0.441	3.650±0.353	n.s
	2018	3.630±0.499	3.514±0.548	4.050±0.212	n.s	3.630±0.499	3.438±0.489	4.200±0.300	n.s
	2019	4.096±0.397	4.072±0.272	4.600±0.141	n.s	4.096±0.713	4.030±0.775	4.566±0.666	n.s

Supplementary Table S17. Primer sequences information and PCRs.

**Gene**	**Region**	**Primers (5 ➡ 3')**	**Primer size (pb)**	**PCR Product size (pb)**	**PCR optimized conditions in °C**	**Références**

MTN R1A	Promoter	**Fragment 1** :<br><br>Fw: GCACAAAAAGAAGCCAAGGA<br><br>Rv: TCAGGTGTCGCACTGTAACC	20<br><br>20	777	Initialisation 94°c 3 min<br>Denaturation 94°c 1 min<br>Annealing 61°c 1 min } 35 cycles<br>Extension 61°c 1 min<br>Final elongation 72°c 10 min	(Cosso, Nehme et al. 2021)<br><br>(Martinez-Royo, Alabart et al. 2012)
	Promoter	**Fragment 2 :**<br><br>Fw: TGTTCCTGGGAGAAGTCTGG<br><br>Rv: CATGCATCAAACCTGGACTG	20<br><br>20	721	Initialisation 94°c 3 min<br>Denaturation 94°c 1 min } 35 cycles<br>Annealing 60°c 1 min<br>Extension 72°c 1 min<br>Final elongation 72°c 10 min	
		**Fragment 3 :**<br><br>FW : GGCCCTAACCCATGTTTTCT<br><br>Rv: CTCCCACTCTGTTCCCTGAA	20	1157	Initialisation 94°c 3 min<br>Denaturation 94°c 1 min<br>Annealing 54°c 1 min } 35 cycles	

	Exon2/3' UTR		20		Extension 72°c 1 min<br><br>Final elongation 72°c 10 min	
